# Occupations and their impact on the spreading of COVID-19 in urban communities

**DOI:** 10.1038/s41598-022-18392-5

**Published:** 2022-08-18

**Authors:** Marian-Gabriel Hâncean, Jürgen Lerner, Matjaž Perc, Iulian Oană, David-Andrei Bunaciu, Adelina Alexandra Stoica, Maria-Cristina Ghiţă

**Affiliations:** 1 Department of Sociology, University of Bucharest, Panduri 90-92, 050663 Bucharest, Romania; 2 Department of Computer and Information Science, University of Konstanz, 78457 Konstanz, Germany; 3 Human Technology Center, RWTH Aachen University, 52062 Aachen, Germany; 4 Faculty of Natural Sciences and Mathematics, University of Maribor, Koroška cesta 160, 2000 Maribor, Slovenia; 5 Department of Medical Research, China Medical University Hospital, China Medical University, Taichung, 404332 Taiwan; 6 Alma Mater Europaea, Slovenska ulica 17, 2000 Maribor, Slovenia; 7 Complexity Science Hub Vienna, Josefstädterstraße 39, 1080 Vienna, Austria

**Keywords:** Statistical physics, Complex networks

## Abstract

The current pandemic has disproportionally affected the workforce. To improve our understanding of the role that occupations play in the transmission of COVID-19, we analyse real-world network data that were collected in Bucharest between August 1st and October 31st 2020. The data record sex, age, and occupation of 6895 patients and the 13,272 people they have interacted with, thus providing a social network from an urban setting through which COVID-19 has spread. Quite remarkably, we find that medical occupations have no significant effect on the spread of the virus. Instead, we find common transmission chains to start with infected individuals who hold jobs in the private sector and are connected with non-active alters, such as spouses, siblings, or elderly relatives. We use relational hyperevent models to assess the most likely homophily and network effects in the community transmission. We detect homophily with respect to age and anti-homophily with respect to sex and employability. We note that, although additional data would be welcomed to perform more in-depth network analyses, our findings may help public authorities better target under-performing vaccination campaigns.

## Introduction

The complexity of today’s society imposes difficulties in predicting and controlling the spread of epidemics^1^. For example, the coronavirus disease 2019 (COVID-19) transmission has been affected by critical factors such as human mobility flows^2,3^, sociodemographic characteristics of the human populations^4,5^, and the structure of social interactions^6^. Notably, researchers have long acknowledged that the spread of diseases has a networked structure: infections move from one individual to another, patterned by human connections^7^. Therefore, many studies have aptly brought forward network science to overcome many of the challenges imposed by the spreading processes of infections^8,9^. Understandably, scientists have employed network theoretical insights, approaches, and measurements^10^ to understand and predict infectious diseases (epidemic outbreaks^11^, spreading of infections, or design immunization strategies^12^). Consequently, our paper employs tools procured from network science to examine the role of occupations and work environments in patterning the circulation of COVID-19.

COVID-19 infection transmission occurs in various probabilities between the individuals embedded in social networks (via direct close contact through aerosols or droplets)^13^. Generally, COVID-19 transmissibility may be understood and modelled by exploring the topology of the networks carrying the infection^14^. For instance, individual infectiousness is often highly skewed, illustrating asymmetric individual-level network degree distributions or super-spreading^15^. Thus, the configurations of social networks significantly impact the relative hazard of infection (a person is tested positive)^6^. Simultaneously, the attributes of the individuals also play an essential role in the disease diffusion. Therefore, we advance statistical models blending network-related variables (e.g., popularity, reciprocation, degree-centrality, etc.) and individual features (such as occupational features) as predictors.

Current evidence suggests that the COVID-19 disease is not uniformly distributed across occupational groups and, implicitly, across the members of a community^16^. Occupational inequalities in exposure to COVID-19 infections mark an unequal distribution of infection cases^17^ across various groups of employees. In the workplace settings, the workers’ degree of physical proximity to their colleagues and customers^18^ has been used to gauge the risk of coronavirus exposure. Based on medical records, some researchers have shown that occupations with a high testing frequency (health personnel, teachers) have a low frequency of positive tests^19^. Remarkably, the existing studies addressing the occupational risk to COVID-19 infections are not built on direct data on infection rates at occupational level^18^ or are only following case studies^20^. Strikingly, to our knowledge, studies to estimate the impact of occupations or work settings in COVID-19 transmission throughout social networks at the community level are missing. In this paper, we analyse real-world individual-level COVID-19 transmission network data, officially recorded, in Bucharest (Romania), between August 1st and October 31st (2020), by the Department of Public Health Bucharest. We use this rare set of network data to assess whether individuals’ status in the labour market (whether they work) and supra-individual factors such as work environment (where they work) affect COVID-19 human-to-human network spreading in urban communities. We apply a new family of statistical models (relational *hyperevent* models^6,21–24^) to specify and estimate the relative hazard that an infected individual nominates a set of social contacts (that is alters whom someone previously interacted with) as potential infection origin, given his/her occupation and work setting. We control for covariates (sex and age) and various network effects (network configurations) in these models. We find that medical occupations have no significant effect on the community spread of the virus. Instead, we note common transmission chains to start with infected individuals who hold jobs in the private sector, and who are connected with non-active alters, such as spouses, siblings, or elderly relatives. In these chains, we also observe that people tend to nominate peers of similar age, females rather nominate males, and active people are inclined to nominate non-active individuals (children, university students, pensioners).

### The relevance of COVID-19 network studies for vaccination strategies

Understanding the role of occupations and workplace settings in the network-wise spread of COVID-19 may have several implications. First, it informs decision-makers to tailor the vaccination campaign deployments better. Second, it contributes to the discussions on estimating the differential occupational risk of COVID-19, given the current scarcity of direct empirical data. Third, it may support managers in organizations to decide whether to bring the employees back to work or allow “work from home policies” (where applicable) during emerging waves with high transmission potential. Fourth, analysing real-world COVID-19 transmission network information may support the development of new network vaccination models.

Public authorities have implemented large-scale non-pharmaceutical interventions (NPIs) to contain the virus spread^25^. These measures (e.g., travel bans, social (physical) distancing, school closure, remote work, lockdowns) have been essential for fighting against the pandemic^26^. Expectedly, NPIs significantly affected the structure of human interactions^27^ and, subsequently, reduced disease transmission^28^. Unfortunately, NPIs have come with substantial social, economic, and health costs. NPIs seem to have differently impacted the labour market (regarding unemployment and job classes)^18^ and ethnic groups^16^, creating new social vulnerabilities or deepening old ones^17^. Additionally, recent evidence has stressed that remote work is possible for only a limited number of jobs (this number does not exceed 40%^29^). Given the challenges raised by the NPIs and the tremendous pressures for fully re-opening the economies, a massive global interest coagulated around the possibility of vaccination^30^. Immunization by vaccination is expected to be the most important intervention for the containment of the COVID-19 pandemic. Recent evidence claims that COVID-19 infections are less likely in vaccinated people^31^. However, (herd) immunization through vaccination needs time to unfold due to vaccine hesitancy, supply-chain obstacles, and provider shortages^32^. To achieve global vaccine immunity, national governments and international bodies need to address various challenges. Namely, ensuring enough doses are available worldwide and equitably distributed, securing access to vaccines for vulnerable populations, and implementing an effective deployment of COVID-19 vaccines^33^.

Mass vaccination has proved efficient in achieving herd immunity for the case of many infectious diseases (meningitis, influenza, yellow fever)^34^. Nevertheless, vaccine hesitancy or opposition represents a severe threat to mass vaccination campaign deployments. The interplay between behaviour dynamics and social network topology may yield essential effects on the diseases spread^35^. Actually, scientists and practitioners have documented and developed alternative strategies to mass vaccination using either observational studies or simulations^7^. One of these strategies is the network vaccination^36^: the individuals who should be immunized are not at random but selected by structural (topological) properties of the social networks. Specifically, researchers have advanced various variants of network vaccination: targeted vaccination (immunization schemes based on the nodes’ connectivity^37^), acquaintance vaccination (the friends of a randomly selected set of individuals are vaccinated^38^), random walk vaccination (immunization is performed by randomly visiting a social network, especially in assortatively mixed networks^39^), or common acquaintance vaccination (the common friends of randomly selected individuals are immunized^40^).

Network vaccination strategies may represent an alternative solution to mass vaccination, especially for communities or countries wherein the immunization coverage rate by the vaccine (the percent of the target population that has received the last recommended dose) is below the projected target. An example is Romania, an Eastern European country accounting for 4.3% of the EU-27 population, i.e., 19.4 million (2019), and having a population density of 83 per square kilometres (2018). As of July 21st, 2022, Romania was next to last among EU/EEA countries concerning full vaccination uptake (the completion of the primary vaccination course), with 42.3% of the population (≥ 18-year-old)^41^. A similar situation was reported for Bucharest, the country's capital: the most recent official reports show that, within the adult population, the full vaccination uptake was 34.9% (as of August 5th, 2021), and the partial vaccination was 49.6% (as of February 5th, 2022)^42^. Hence, understanding the early stage of COVID-19 spread through social networks may inform local authorities to better respond to problematic situations at later stages (e.g., the low levels of vaccination).

## Results

Our findings show that individuals’ status in the labour market and the work environment affect the COVID-19 spreading in the community. We have records of 6895 patients (referees) and their social contacts (13,272 referrals). Table 1 renders descriptive statistics for the variables used in the statistical models: the type of the sector (public/private), medical sector affiliation (yes/no), status in the labour market (active or not active), and age classes with relevancy for the labour market (minors, adults, and pensioners).

**Table 1:** Descriptive statistics for variables used in the statistical modelling.

Categories by variables of interest*	Referees	Referrals
***Unemployed**** (including unemployable)*		
Children (< 7-year-old)	107 (1.6%)	1388 (10.5%)
Pensioners (retired)	537 (7.8%)	1029 (7.8%)
University students	22 (0.3%)	2 (< 0.0%)
School students	331 (4.8%)	2345 (17.7%)
No formal job at the moment of the interview	49 (0.7%)	5 (< 0.0%)
*Total unemployed (including unemployable)*	1046 (15.2%)	4769 (36.0%)
Valid data (employed and unemployed)	2225 (32.3%)	4907 (37.0%)
Missing data	4670 (67.7%)	8365 (63.0%)
Total referees	6895 (100.0%)	13,272 (100.0%)
Employed by type of sector**		
Private	757 (11.0%)	96 (0.7%)
Public	422 (6.1%)	42 (0.3%)
*Total employed by type of sector*	1179 (17.1%)	138 (1.0%)
Valid data (employed and unemployed)	2225 (32.3%)	4907 (37.0%)
Missing data	4670 (67.7%)	8365 (63.0%)
Total referees	6895 (100.0%)	13,272 (100.0%)
***Working in the medical sector***		
Yes	175 (2.5%)	22 (0.2%)
No	1013 (14.7%)	120 (0.9%)
*Total medical sector affiliation (Yes* + *No)*	1188 (17.2%)	142 (1.1%)
Unemployed and unemployable	1046 (15.2%)	4769 (36.0%)
Valid data	2234 (32.4%)	4911 (37.0%)
Missing data	4661 (67.6%)	8361 (63.0%)
Total referees	6895 (100.0%)	13,272 (100.0%)
***Active for the labor market*** (employed and employable)***		
Yes	5896 (85.5%)	8432 (63.5%)
No	997 (14.5%)	4764 (35.9%)
*Total active for the labor market (Yes and No)*	6893 (≈100.0%)	13,196 (99.4%)
Valid data	6893 (*≈*100.0%)	13,196 (99.4%)
Missing data	2 (< 0.1%)	76 (0.6%)
Total referees	6895 (100.0%)	13,272 (100.0%)
***Age groups (relevant categories for the labor market)***		
Minors (< 18 y.o.)	436 (6.3%)	3733 (28.1%)
Adults	5920 (85.9%)	8434 (63.5%)
Pensioners (retired)	537 (7.8%)	1029 (7.8%)
*Total age groups*	6893 (≈100.0%)	13,196 (99.4%)
Valid data	6893 (*≈*100.0%)	13,196 (99.4%)
Missing data	2 (< 0.1%)	76 (0.6%)
Total referees	6895 (100.0%)	13,272 (100.0%)

*Within a total of 20,167 unique cases (6895 referees and 13,272 referrals), 7130 are unique cases with full information (2223 referees and 4907 referrals) on the variables of interest (age, sector, medical sector affiliation, and status on the labor market). **Public = state-owned organizations, *Private* = private companies. ***Employable = not working but eligible to work.

In our dataset, most of the referees (referrals) are working in the private sector. People holding jobs in the medical sector (doctors, nurses, medical managers, etc.) account for 2.5% (referees) and 0.2% (referrals) in the total sample. A large majority of the referees (85.5%) and referrals (63.5%) are “active in the labour market” (either employed or in search for work, that is employable). We consider minors (less than 18 years old), university students, and pensioners to be not active (neither working nor in search of work). The average age of the referees is 40.6 (S.D. = 15.2, Min = 0, Max = 97, n = 6,892) while the average age of the referrals is 33.6 (S.D. = 21.0, Min = 0, Max = 95, n = 13,196). We follow the International Standard Classification of Occupations (ISCO-08) structure^43^ and give details about the jobs of the referees and referrals as Supplementary Information (Tables S1–3).

Table 2 reports the findings corresponding to the fit statistical models: *the covariate model* (the model specified solely based on node-level covariates), *the network model* (the model that includes only network-level variables), and *the joint model* (the model including both covariate and network effects). A detailed description of these models is available in the "Methods" section.

**Table 2:** Relational hyperevent model assessment: comparing the covariate, the network, and the joint models.

	Covariate model		Network model			Joint model	
	Exp(Coef) 95% CI	Coef (SE)	Exp(Coef) 95% CI	Coef (SE)		Exp(Coef) 95% CI	Coef (SE)
Age difference	0.498 (0.460–0.540)	− 0.697 (0.041) ***				0.521 (0.477–0.568)	− 0.652 (0.045) ***
Avg. age of the referrals	1.004 (0.950–1.062)	0.004 (0.029)				1.020 (0.960–1.083)	0.019 (0.031)
Age of the referee	1.746 (1.632–1.868)	0.557 (0.034) ***				1.612 (1.496–1.737)	0.477 (0.038) ***
Sex difference (males = 1, females = 2)	2.023 (1.892–2.164)	0.705 (0.034) ***				1.873 (1.738–2.020)	0.628 (0.038) ***
Sex of the referrals	1.091 (1.019–1.167)	0.087 (0.035) *				1.113 (1.028–1.204)	0.107 (0.040) **
Sex of the referee	1.047 (1.006–1.089)	0.046 (0.020) *				1.040 (0.997–1.084)	0.039 (0.021)
Referral in public sector	0.707 (0.601–0.832)	− 0.347 (0.083) ***				0.753 (0.637–0.890)	− 0.284 (0.085) ***
Referee in public sector	0.965 (0.937–0.995)	− 0.035 (0.015) *				0.957 (0.927–0.987)	− 0.044 (0.016) **
Referral in medical sector	0.835 (0.697–0.998)	− 0.181 (0.092) *				0.878 (0.738–1.045)	− 0.130 (0.089)
Referee in medical sector	1.000 (0.974–1.027)	0.000 (0.014)				0.997 (0.969–1.025)	− 0.003 (0.014)
Active workforce difference (Active = 1, non-active = 0)	1.182 (1.078–1.296)	0.167 (0.047) ***				1.253 (1.128–1.392)	0.226 (0.054) ***
Active referrals	0.430 (0.391–0.474)	− 0.843 (0.050) ***				0.493 (0.440–0.553)	− 0.707 (0.058) ***
Active referees	2.286 (2.117–2.468)	0.827 (0.039) ***				2.276 (2.093–2.475)	0.822 (0.043) ***
Individual contact popularity (in-degree of the referral)			0.006 (0.003–0.011)	− 5.139 (0.309) ***		0.008 (0.004–0.014)	− 4.878 (0.306) ***
Joint contact popularity			1.116 (1.081–1.152)	0.110 (0.016) ***		1.110 (1.063–1.158)	0.104 (0.022) ***
Reciprocation			1.131 (1.111–1.152)	0.123 (0.009) ***		1.111 (1.088–1.134)	0.105 (0.010) ***
In-degree of the referee			0.229 (0.195–0.271)	− 1.472 (0.084) ***		0.307 (0.258–0.365)	− 1.182 (0.088) ***
Out-degree of the referral			0.056 (0.038–0.082)	− 2.880 (0.195) ***		0.070 (0.048–0.103)	− 2.655 (0.194) ***
Nominations among contacts			1.077 (1.062–1.092)	0.074 (0.007) ***		1.081 (1.066–1.097)	0.078 (0.007) ***
Shared referee			1.110 (1.089–1.131)	0.104 (0.010) ***		1.104 (1.084–1.124)	0.099 (0.009) ***
AIC	9375.042		8592.547			6293.281	
Num. events	1179		1179			1179	
Num. obs	1,250,975		1,250,975			1,250,975	

***p < 0.001; **p < 0.01; *p < 0.05; *p* < 0.1. The Table provides the hazard ratios (Exp(Coef.)), their 95% confidence intervals, the effects (Coef.) and the *p* value for each variable included in the models.

Referring to the joint model, we find that age has a positive effect on the probability of appearing as a referee (*age of the referee*). That means older persons have a higher risk of being tested positive (HR = 1.612, 95%CI [1.496–1.737]). On the other hand, age has no significant effect on the probability of being nominated as a contact (*average age of the referrals*) (HR = 1.020, [0.960–1.083]). However, the average age difference between the referee and the referrals has a decreasing effect (*age difference*) (HR = 0.521, [0.477–0.568]). This finding points to the age-homophily of contact-nomination events: older positive cases tend to nominate older contacts, and younger positive cases tend to nominate younger contacts. We also find that females are more likely to be positive cases (*sex of the referee*) only when we do not control for network effects, and more likely to be nominated as contacts (*sex of the referrals*) (HR = 1.113, [1.028–1.204]). Nevertheless, controlling for other effects, we find that it is anti-homophily with respect to sex (*sex difference*) (HR = 1.873, [1.738–2.020]). That means females are more likely to nominate males and vice versa. This finding may suggest that spouses nominate their partners as contacts.

Further, persons working in the public sector are less likely to be tested positive (*referee in public sector*) (HR = 0.957, [0.927–0.987]) and less likely to be nominated as contacts (*referral in public sector*) (HR = 0.753, [0.637–0.890]). The covariate model shows that persons working in the medical sector are again less likely to be nominated as contacts (*referral in medical sector*) (HR = 0.835, [0.697–0.998]); this variable has no significant effect on the probability of being tested positively (*referee in medical sector*) (HR = 1.000, [0.974–1.027]). It is noteworthy that there is a substantial overlap between the medical and public sectors. Note that our models (*the covariate* and *joint models*) also control for which person is active. We find that active persons in the workforce are more likely to be tested positive (*active referees*) (HR = 2.276, [2.093–2.475]) but less likely to be nominated as a contact (*active referrals*) (HR = 0.493, [0.440–0.553]).

Moreover, we find anti-homophily with respect to the *activity* (*active workforce difference*). Precisely, active persons are more likely to nominate non-active persons (HR = 1.253, [1.128–1.392]). In summary, the findings reported in Table 2 suggest that typical contact-nomination events are due to working persons (mainly in the private sector and typically older than average) who are tested positive and then nominate non-working partners of the opposite sex but of similar age as contacts.

The estimates for the *network model* are in accord with similar previous studies^6^. In the network and joint models, we use several variables: the *in-degree of the referee* and *referral,* and the *out-degree of the referral*. These are significantly negative. Notably, the *out-degree of the referee* cannot be taken as a variable. As we mention in the "Methods" section, no person is tested positively more than once, and we remove the positives from the pool of possible future positives so that the *out-degree of the referee* is necessarily zero). These effects control for the property that only persons, who appear as a referee or as a referral (or as both), are included in our sample. Once this event has happened, their probability of participating in a future contact-nomination event decreases.

The positive coefficients of the network effects (the network and joint models, in Table 2) reveal that persons who jointly appeared in a past contact-nomination event (in any role) are more likely to co-appear in a future event. Specifically, suppose two persons A and B have been co-nominated as contacts in the past; they are more likely to be co-nominated by another positive case in the future (positive effect of the *joint contact popularity*) (HR = 1.110, [1.063–1.158]). Moreover, it is more likely that A nominates B (positive effect of the *shared referee*) (HR = 1.104, [1.084–1.124]). Also, suppose a person A has been tested positively and has nominated another person B as a contact. In that case, B is more likely to nominate A as a contact (positive effect of the *reciprocation*) (HR = 1.111, [1.088–1.134]), and it is more likely that a third person C co-nominates A and B as contacts (positive effect the *nominations among contacts*) (HR = 1.081, [1.066–1.097]).

Finally, Table 2 reports *the joint model* (including both covariate and network effects). We find that qualitatively almost no effects change when the two sets of predictors are combined. The strongest difference is that persons working in the medical sector are no longer significantly less likely to be nominated as contacts. Moreover, females are still more likely to be positive cases—but this finding is, now, only significant at the 10% level.

Table 2 displays the model hazard ratios, their 95% confidence intervals, coefficients (*Coef.*) and their standard errors (*SE*) in brackets side-by-side. We find that the network-only model has a better fit than the covariate-only model (lower AIC implying better model fit) but that the combined model has the best fit of all. Thus, unless otherwise, the aforementioned hazard rations are subtracted from the joint model (Table 2). (Details about the models are available in the Supplementary Information, Tables S4–6).

## Discussion

Our findings suggest that individuals’ status in the labour market and the work environments significantly affect the transmission of COVID-19 in Bucharest, Romania. Namely, more active older persons (mainly in the private sector) are tested positive. They nominate as contacts people of similar age and of opposite sex who are relatively not active (or particularly not in the public sector). Medical occupations have no significant effect when controlling for network effects. Otherwise, people with jobs in the health-care sector are significantly less likely to be nominated as contacts. Furthermore, people working in the public sector are less likely to be *positives* and less likely to be *contacts* than those in the private sector. We also find that females are slightly more likely to be positives and contacts (and that anti-homophily in contact-nomination is rather strong, *i.e.,* probably spouse nominates spouse). The base effects have to be understood together with the other variables. If we assume that more males are active—and active persons are much more likely to be positives—then the positives are more likely male. Just if two individuals are both active and one is male and the other female, then the female is slightly more likely to be positive than the male.

Scientists have given health-care workers (HCWs) special attention given reported nosocomial spread and depletion of staff numbers^44^. Evidence from the early stage of the pandemic has generally indicated HCWs as the occupational group with the highest risk of exposure to COVID-19 infections^45^. Later findings may seem, however, contradictory. Some studies claim that people working in the medical sector hold a pivotal role in spreading the disease^2^. Others, on the contrary, do not report strong evidence for patient-to-HCWs (or HCWs-to-patients) transmission chains. For instance, a large observational study performed in the Netherlands^46^ has claimed that childcare, education, and health-care workers display lower test positivity than public transport workers, driving instructors, or hairdressers. A cross-sectional study performed on workplace data in Qatar has attributed the lowest positivity rate to the health-care workplace settings^47^. Structured interviews and whole-genome sequencing of SARS-COV-2 performed on a sample of 1,497 HCWs, in the south of the Netherlands^48^ did not support widespread nosocomial transmissions as the infection source. Inversely, this study has indicated that the infections among HCWs were caused by community transmission. Additionally, a lack of strong evidence for patient-to-HCW transmissions has been reported in a retrospective cohort study among Massachusetts HCWs^49^. Our study is consistent with these aforementioned investigations and others^50^. Precisely, we did not find a significant effect for medical occupations in the virus transmission chains.

Existing studies are intrinsically affected by various factors such as the pandemic stage (when the data have been collected), the employed research methods, the cultural variation. As cross-country comparative research^51^ or longitudinal studies^52^ are rare, comparing the cumulated evidence is challenging. Some scientists have advocated that the disparities among occupations in terms of infection rates may be directly linked to the nature of jobs (exposure to disease and proximity to colleagues and clients) and their surrounding settings (the density of daily contacts, the ventilation of the rooms)^16^. Clear evidence and a large accord among researchers are, however, still missing. Occupations greatly vary from multiple perspectives^52^: fluctuations in the number of performed tests, availability of protective equipment, organizational procedures, etc. Our study emphasizes the importance of active (working) people in the COVID-19 transmission chains, which is in line with previous evidence reported, for instance, in Poland^53^.

We hope to contribute to the currently active debate concerning the impact of occupations on the COVID-19 spreading in the community. We document transmission chains wherein people holding jobs in the private sector (COVID-19 positive cases) are connected to non-active alters of different sex (likely their spouses). Further research is required to confirm whether these transmission chains directly connect working settings to domestic households. Additional work is needed to have a more in-depth understanding of why the private sector has sustained a resurgence in the transmission of novel coronavirus disease. The diverseness of the private sector may explain different practices concerning the application of the COVID-19 preventive measures. While, conversely, the uniformity of the public sector may have been more efficient in implementing measures for containing the virus spread. In this respect, our results may be deemed important to the debate on whether COVID-19 should be classified as an occupational disease.

Our paper displays at least two elements of originality. To our knowledge, this is the first study to address the role of occupations in the transmission of COVID-19 in an urban community by using network data as empirical evidence. Further, we use an innovative, recently developed family of statistical modelling (relational hyperevent modelling). By applying this methodology, we can gain further insights into the mechanisms of the virus spread. Namely, we can assess the impact of people’s attributes (e.g., occupations) and the effect of the shape of their social networks.

Some limitations of this study are worth noting. Firstly, the observed human-to-human transmission network covers a time window of only three months (August 1st– ctober 31st, 2020). This time interval corresponds to a moderate level of stringency measures in Romania^54^ (e.g., school reopening, organization of political elections, lack of internal movement restrictions, remote work was not mandatory). Lacking data about the earlier part of the pandemic prevents us from assessing its impact on the time window analysed here. Future work may reveal whether and how official measurements affect the role of occupations and work settings in the virus spreading. Secondly, we analyse epidemiological information collected by the public health authorities (i.e., only the officially recorded cases). The corresponding dataset contains a consistent share of missing data, preventing us from performing a more diverse statistical analysis. Authorities should consider improving their data collection tools through future collaborations with academia. Also, our results are relevant only for the “population” of officially COVID-19 confirmed cases. Given the complexity of the COVID-19 community spread, we avoid reckoning representativeness for “all” COVID-19 cases that may have covertly existed at the time. Despite these limitations, our findings may be generalized to communities with similar occupation, age, and sex distributions, anti-COVID-19 measures, and similar cohabitation behaviour: similar household socio-demographics^6^. Additionally, our findings may be informative for future research on different waves, stringency measures and virus variants.

We build on previous studies claiming COVID-19 cases are not uniformly distributed across occupational groups. Yet, we move forward by addressing occupations, not in isolation, but as interconnected. Namely, we statistically mix people’s attributes with their network position in the transmission of COVID-19. That allows unveiling the role of occupations within the networked circulation of the virus. For the time interval that we analysed, we did not find a significant effect for medical occupations in the virus transmission chains. Probably because, at this stage, healthcare workers were better trained to use Personal Protective Equipment effectively, had more supplies, and had been largely infected in the previous pandemic stages. Also, we learn that people working in the private sector exhibited a pivotal role in virus diffusion. These findings may suggest that the impact of occupations on the networked circulation of the virus changes from one wave to another. All things considered, modelling the COVID-19 networked transmission as an occupational and not as an individual problem may provide new insights for public health authorities and company managers. Namely, it may have various implications: for the ongoing under-performing vaccination campaigns in Bucharest (Romania) and other related urban communities worldwide, for future studies focused on different stages of the pandemic, and for managers planning remote work policies within organizations.

## Methods

In this observational study, we use data that illustrate how COVID-19 infections may have spread from one individual to another in Bucharest (Romania) between August 1st and October 31st, 2020. The dataset includes all officially COVID-19 confirmed cases in the indicated time window. We have records for 6895 patients and, also, for the people they interacted with (13,272 social contacts) before the onset of the COVID-19 symptoms (i.e., *Whom did you interact with three days before the first COVID-19 symptoms?*). In our study, the patients are dubbed as *referees* while their nominated contacts, *referrals*. There are 454 actors that appear both as referees and referrals (dubbed as *brokers*). Namely, these individuals appear in the dataset collection as *patients* per se and social contacts for other patients.

Our real-world COVID-19 human-to-human transmission network data^55^ is procured from the Department of Public Health Bucharest (DPHB, Ministry of Health, Romania). DPHB is the local public health authority responsible for officially collecting, recording and reporting evidence on COVID-19 cases. Subsequently, this evidence is used for developing public health policies and interventions. To improve the interpretability of the data and the accompanying results, it should be noted that, at the time of the data collection, vaccination was not in place in Romania. The first vaccinated person was reported on December 27, 2020, according to the Romanian authorities. Consequently, our analysis corresponds to a pre-vaccination context and, therefore, vaccination information is not available in the dataset received from DPHB. The authors of this paper were not part of the official data collection process. Therefore, we cannot provide insights and details about the intricacies of the procedures employed by DPHB to provide the official records of COVID-19 in Bucharest. For this reason, we limit to only acknowledging that we use official COVID-19 pandemic data issued by a local official authority. And, in extension, the epidemiological information includes all the COVID-19 cases officially reported in Bucharest, in the indicated timeframe. Therefore, for accuracy, our results are relevant for this special population of “officially” reported cases and not for “all” COVID-19 cases that may have existed at the community level between August 1st and October 31st 2020. Furthermore, our statistical analysis is limited to official evidence and to the variables available in the dataset that was procured from DPHB. In this respect, other variables which may have been useful for understanding the pandemic context in Bucharest are not accessible to us (e.g., the number of people who tested negative and their socio-demographic profile). Also, we cannot gauge the representativeness of our data as previous studies or official reports on the virus spread in Bucharest (Romania) are not available (e.g., information about the COVID-19 prevalence by occupations).

**Figure 1:**
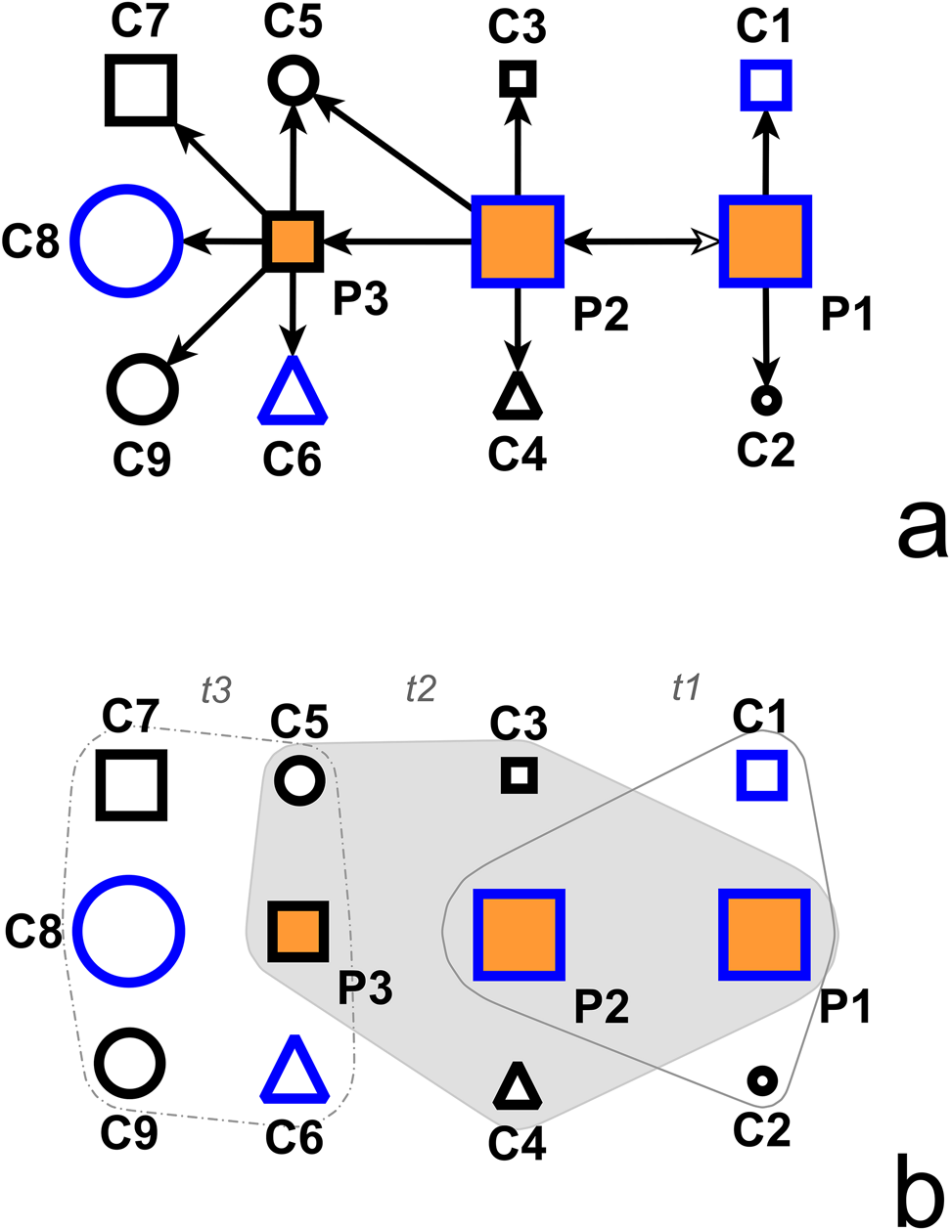
Illustration of a human-to-human COVID-19 transmission network. Positive cases (referees) are in orange, and referrals (contacts) are in white. Blue-bordered nodes mark females while black-bordered mark males. Geometric shapes correspond to occupational classes: squares designate people in the private sector, triangles mark people in the public sector, and circles are not-active individuals (minors, pensioners, university students). Arrows mark the direction of the elicitation process: pointing from the referee (positive case) to the referral (nominated contact). Node size is proportional to age. The image displays (**a**) dyadic and (**b**) hypergraph representations of the transmission network. In Fig. 1b, the hypergraphs (contact-nomination events) are attached time-stamps to show their position in time. Overlapping areas mark nodes belonging to more than one hypergraph.

In our analysis, we use several covariates such as sex (male or female), age, and major occupational groups: the sector type (public or private), whether a person works in the health-care industry (yes/no), and whether they are active or not (pensioners, minors, university students). We derive these major occupational groups from the job information collected by the public health authorities in their epidemiological survey. We employ the International Standard Classification of Occupations (ISCO-08). For instance, the ISCO-08 one-digit structure is: armed force occupations; managers; professionals; technicians and associate professionals; clerical support workers; service and sales workers; craft and related trade workers; plant and machine operators, and assemblers; elementary occupations (see Tables S1–3, Supplementary Information). Remarkably, at the time of the data collection, healthcare workers were not routinely tested as a special occupational group. This situation filters out potential biases due to different existing COVID-19 testing practices by occupation. Additionally, we know the day when a referee was confirmed officially as a positive case. And, we include in the analysis the network configurations embedding the referees and the referrals. Finally, we test in the statistical models these structural configurations as network effects. Figure 1a provides a glimpse of the analysed dataset structure.

Essentially, our data comprises time-stamped contact-nomination events of the form (*t*, *P*, {*C*1, …,* Ck*})*.* This tuple shows that at time *t*, the confirmed positive case *P* (referee) nominates the *k* contacts *C*1, …, *Ck* (referrals) when being interviewed by health authorities. In a nutshell, we estimate the effect of various covariates (including, but not being limited to, occupational classes) on the relative risk of observing these contact-nomination events via Cox proportional-hazard models. There are three fundamentally different types of possible effects, which we interpret in different ways. First, the impact of covariates of the positive case *P* can reveal which persons are more or less likely to be tested positively. Such findings can serve as a proxy for effects that increase or decrease the infection risk. (We recall that our data reveals infections only in a one-sided manner. If a person is tested positive, this indicates that this person is indeed infected—up to the margin of false positives, which is relatively low. However, if a person is not tested positive, this indicates that this person is either not infected or infected but not tested—or is a false negative). Second, the effect of covariates of the contacts *C*1, …, *Ck* can reveal which persons are more likely to be nominated as contacts. Such findings can reveal possible sources of infection (if the positive case got the virus from one of her contacts) or persons who got possibly infected by *P*. Finally, explanatory variables combining covariates of the positive case *P* and her contacts *C*1, …, *Ck*—such as the average age difference between *P* and *C*1, …, *Ck*, or the number of contacts that are in a different occupation class than P—can reveal who is more or less likely to be nominated by whom, giving a more detailed understanding of possible transmission paths. To provide an example of the distinction between these types of effects, we foreshadow the finding that in our data, active persons are more likely to be tested positive, less likely to be nominated as contacts and that contact-nomination ties are more likely between persons of different activity levels (anti-homophilous effect of working activity in contact nominations). In contrast, contact-nomination events reveal homophily with respect to age so that older positive cases are more likely to nominate older contacts and younger positives are more likely to nominate younger contacts.

Estimating the relative risk of contact-nomination events (*t*,* P*, {*C*1, …,* Ck*}) has to take into account that the implied dyadic ties (*P*,* C*1), …, (*P*,*Ck*) are non-independent by design since they are all originating from the same positive case *P*. For instance, a positive case may list here an entire group of work-colleagues as contacts, and it would be invalid to treat them all as independent instances in a proportional hazard model. Relational hyperevent models (*RHEM*) tackle this intrinsic non-independence by treating the entire hyperevent (*t*,* P*, {*C*1, …, *Ck*}) as one single instance whose hazard is specified dependent on covariates that are functions of the entire hyperedge (*P*, {*C*1, …, *Ck*}). In a *hypergraph,* a *hyperedge* constitutes a relation between any number of nodes. In contrast, in a graph, an edge always precisely connects two nodes. We denote as a hyperevent a hyperedge with a time-stamp (which indicates when the interaction takes place)*.* In Fig. 1, we illustrate COVID19 spreading both in the form of a graph and of hypergraphs. Moreover, RHEM can control for various *network dependencies* in contact-nomination data. For instance, if a positive case *P* nominates the contacts *C*1, *C*2, …, then it is likely that *P, C*1, and *C*2 are “close” in some unobserved (latent) social space^56^. In turn, this increases the likelihood that *C*1 nominates *P* and/or *C*2 if *C*1 is interviewed as a confirmed positive case. The previous work^6^ has shown that such network effects are indeed very strong in contact-nomination networks.

We extend the application of RHEM^6^ in two ways. First, we estimate the effect of additional covariates taking into account the major occupational classes of people. Second, we extend the core RHEM by also assessing predictors of the risk of being tested positively. While the previous work^6^ conditioned on the positive case *P* of an observed contact-nomination event (*t*,* P*, {*C*1, …,* Ck*})—and estimated the conditional probability that *P* nominates the contacts *C*1, …,* Ck*, rather than an alternative set of contacts *C*1’, …,* Ck’*—in our models the positive case is also treated as a random variable. Thus, our models seek to explain why a contact-nomination event involves the observed hyperedge (*P*, {*C*1, …,* Ck*}), rather than an alternative hyperedge (*P*’, {*C*1’, …,* Ck*’}) where the alternative positive case *P’* can be different from the observed positive case *P*. The benefit from this model extension is that we can also assess the effect of predictors on the risk of being tested positively—not just the effect of predictors on being nominated as contact. As it turns out in our empirical analysis, some covariates can have a qualitatively different effect on the probability of being tested positively than on the probability of being nominated as contact.

Technically, model estimation proceeds by comparing observed events (*t*,* P*, {*C*1, …,* Ck*}), with alternative hyperedges (*t*,* P*’, {*C*1’, …,* Ck’*}), on which contact-nomination events could have been observed but are not (“non-events” or “censored instances”) in a CoxPH model. Since the number of possible hyperedges is exponential in the number of nodes of the network, we apply case–control sampling^57,58^ by drawing 1000 non-events for each observed event uniformly at random. The sampling of non-events and computation of explanatory variables is made with the software *eventnet*^59^. Parameter estimation is done with the function coxph in the R package *survival*^60^.

We fit three main models. A model that is based only on the covariates age, sex, active, public, and medical (*the covariate model*), a model specified only via network effects (*the network model*), and a joint model that combines all explanatory variables (*the joint model*). The only variable for which we imputed missing values is sex. We have chosen to do so since this variable is not related to the core objectives of this article and since the distribution of sex in our sample (more precisely, in the sub-sample for which this variable is not missing) is roughly equal to the distribution in the entire population of Bucharest (53% females in our sample versus 52.3% in the entire population). Thus, we believe that imputed values (setting missing sex variables to 53% female) have a minor effect on our analysis. In parallel, we also fit our statistical models using another procedure of imputation for missing data on sex (i.e., this time, we randomly imputed the missing data; see Table S7 in the Supplementary Information). The two imputation techniques only slightly (or insignificantly) influence the estimates for our predictors of interest (their interpretation remains the same), which marks a sign of robustness in our findings.

On the other hand, sex was missing surprisingly often in our study and, therefore, would have resulted in a disproportionate reduction of instances. Perhaps this variable was not deemed necessary by health authorities. Variables characterizing the occupational classes were also frequently missing—but since these are related to the core research questions, we did not impute values.

We drop an instance from the analysis if it has a missing value in one (or several) of the variables (excluding sex for which we use imputed values). Even though different models are specified by different sets of variables and thus could be fitted to varying sets of instances, we drop an instance from all models if it has any missing value for any variable. Fitting models to the same set of instances ensures that we can compare model fit via information criteria. We standardized all variables to mean zero and standard deviation equal to one to get standardized coefficients.

Although theoretically, a person could be tested positive more than once in the observation period of three months, this did not happen to any instance. We, therefore, take this structural feature into account by removing positive cases from the pool of persons that could be positive cases in the future. In other words, once a person experiences a *tested-positive event*, this person is removed from the risk set (the set of persons that could be tested positive) right after the event. However, note that such a positive case remains in the risk set of being nominated as a contact by another positive case afterwards.

Referring to the fit of our statistical models (see Results section, Table 2), we report both the hazard ratios (Exp(Coef)) with their corresponding 95% confidence intervals as well as the coefficients (estimates) and their standard errors (SE). These details are expected to provide rapid insights on the magnitude of the impact and on the behaviour of the predictors of interest.

To clarify the statistical modelling procedure, the unit of analysis in our study is the relational event (contact-nomination event); briefly, the instance wherein a patient nominates at least two contacts. Out of 6895 contact-nomination events, we included 1179 in the analysis. The other events had to be dropped since some covariate that we used in the analysis—and that was not sex (which we imputed)—was missing for either the referee or for so many contacts that fewer than two contacts were left. (In our analysis, some of the statistics use the difference in covariates among the contacts and for fewer than two contacts differences are undefined. We also dropped contact-nomination events in which just one contact has been nominated, for the same reason. Our covariates are age, public sector (sector type), medical sector, and active (status on the labour market). By “active” we refer to people who are both employed and employable (no job but eligible to work). The sector type variable makes a clear distinction between state-owned organizations and private companies. Additionally, we worked with a dataset in which we have records for 6895 unique referees and 13,272 unique referrals. Out of these 20,167 unique cases (referees and referrals), we can identify 7130 unique cases with full information on the variables of interest (age, sector type, medical sector affiliation, and status on the labour market). That is 2223 unique referees and 4907 unique referrals—with full information. Even if our unit of analysis is represented by the relational events (i.e., n = 1179), details about the number of cases (with and without full information on the variables of interest) may be deemed relevant for contextualizing the statistical analysis.

It is noteworthy that, in our study, we do not model the incidence of COVID-19 in the population (Bucharest, Romania). That is our statistical models (with our data) cannot explain who (among the population) will be COVID-19 infected earlier, who later, and who not at all. Instead, our models condition on an infection event and then explain which infected person (referee) is linked to which set of contacts (referrals). Hence, our models explain the relation: who is linked to whom via case-contact relations. Our models thus can explain possible pathways of infection (between cases and their contacts). Overall, we have records of 20,167 unique cases (among which 6,895 are infected people and 13,272 are their contacts). Within our data, when we observe a contact-nomination event (A,{B,C,D}), our models seek to explain: (1) why is A the infected case (rather than another person in our sample) and (2) why does A nominate the set of contacts {B,C,D} (and not another set of possible contacts in our sample). Our analysis is necessarily restricted to our sample of confirmed positive cases and their contacts. But the case-contact links among this sample of persons are considered random and are explained by our model.

### Ethical approval

This study received ethical approval (Decision No. 1, from September, 1, 2020) from the Ethics Committee of the Department of Sociology (University of Bucharest). The conducted research was performed according to the Ethics Code of the University of Bucharest. This retrospective study was performed on anonymized data provisioned by the Department of Public Health Bucharest (the Romanian Ministry of Health) based on the request address No. 14870 (August 18th, 2020) made by the University of Bucharest. The Department of Public Health Bucharest (the Romanian Ministry of Health) provided the data based on approval No. NT3054E (August 28th, 2020).

## Supplementary Information

Supplementary Information.

## Data Availability

The dataset analysed in the current study is available in the Zenodo repository, https://doi.org/10.5281/zenodo.6899738.
