# Supplementary material for: Occupations and their impact on the spreading of COVID-19 in urban communities
Source: Sci Rep. 2022 Aug 18;12:14115. doi: 10.1038/s41598-022-18392-5 (PMC9387884; doi:10.1038/s41598-022-18392-5)
Supplement: Supplementary file 1 — Supplementary Information. [file 41598_2022_18392_MOESM1_ESM.pdf]

Supplementary information for
Occupations and their impact on the spreading of COVID-19 in urban communities

Marian-Gabriel Hâncean[1]*, Jürgen Lerner[2,3], Matjaž Perc[4-7], Iulian Oană[1], David-Andrei Bunaciu[1], Adelina Alexandra Stoica[1], Maria-Cristina Ghiță[1]

[1] Department of Sociology, University of Bucharest, Bucharest, Panduri 90-92, 050663, Romania.
[2] Department of Computer and Information Science, University of Konstanz, 78457, Konstanz, Germany.
[3]Human Technology Center, RWTH Aachen University, 52062, Aachen, Germany.
[4] Faculty of Natural Sciences and Mathematics, University of Maribor, Koroška cesta 160, 2000 Maribor, Slovenia.
[5] Department of Medical Research, China Medical University Hospital, China MedicalUniversity, Taichung 404332, Taiwan
[6] Alma Mater Europaea, Slovenska ulica 17, 2000 Maribor, Slovenia.
[7] Complexity Science Hub Vienna, Josefstädterstraße 39, 1080 Vienna, Austria.
* email: gabriel.hancean@sas.unibuc.ro

Table S1A General presentation of referees' jobs by ISCO-08 one digit categories and sector type.

ISCO-08 one digit	Patients (referees)	Employed in the public sector	Employed in the private sector	Missing data about type of sector
Armed forces occupations [0]	19 (0.3%)	19	0	0
Managers [1]	25 (0.4%)	1	20	4
Professionals [2]	211 (3.1%)	122	89	0
Technicians and associate professionals [3]	30 (0.4%)	14	16	0
Clerical support workers [4]	29 (0.4%)	7	22	0
Service and sales workers [5]	85 (1.2%)	37	48	0
Craft and related trades workers [7]	18 (0.3%)	4	14	0
Plant and machine operators, and assemblers [8]	13 (0.2%)	1	12	0
Elementary occupations [9]	2 (<0.0%)	0	2	0
Total (valid information)	*432 (6.3%)*			
Active - household (not formally working)	10 (0.1%)	*Not applicable*	*Not applicable*	*Not applicable*
Active – maternity / medical leave	23 (0.3%)	1	4	18
Active - unemployed	38 (0.6%)	*Not applicable*	*Not applicable*	*Not applicable*
Employee with no formal job - unknown group	1 (<0.0%)	*Not applicable*	*Not applicable*	*Not applicable*
Employee - unknown group	796 (11.5%)	216	530	50
Not Active - child	107 (1.6%)	*Not applicable*	*Not applicable*	*Not applicable*
Not Active - pensioner	537 (7.8%)	*Not applicable*	*Not applicable*	*Not applicable*
Not Active – university student	22 (0.3%)	*Not applicable*	*Not applicable*	*Not applicable*
Not Active - school student	331 (4.8%)	*Not applicable*	*Not applicable*	*Not applicable*
Missing Data	*4,598 (66.7%)*			
Grand total	*6,895 (100.0%)*			

We give ISCO-08 one-digit occupation codes in squared brackets and report how many people are in each occupational category (absolute and relative frequencies). The last three columns show how many referees, from each occupational category, are employed in the public or private sector (row-wise absolute frequencies).

Table S1B General presentation of referrals' jobs by ISCO-08 one digit categories and sector type.

**ISCO-08 one digit**	**Social contacts (referrals)**	**Employed in the public sector**	**Employed in the private sector**	**Missing data about type of sector**
Armed forces occupations [0]	2 (<0.0%)	2	0	0
Managers [1]	0 (<0.0%)	0	0	0
Professionals [2]	31 (0.2%)	13	18	0
Technicians and associate professionals [3]	2 (<0.0%)	1	1	0
Clerical support workers [4]	4 (<0.0%)	0	4	0
Service and sales workers [5]	13 (0.1%)	3	10	0
Craft and related trades workers [7]	3 (<0.0%)	0	3	0
Plant and machine operators, and assemblers [8]	2 (<0.0%)	0	2	0
Elementary occupations [9]	2 (<0.0%)	0	2	0
Total (valid information)	*59 (0.4%)*			
Active - household (not formally working)	1 (<0.0%)	*Not applicable*	*Not applicable*	*Not applicable*
Active – maternity / medical leave	4 (<0.0%)	0	0	4
Active - unemployed	3 (<0.0%)	*Not applicable*	*Not applicable*	*Not applicable*
Employee with no formal job - unknown group	1 (<0.0%)	*Not applicable*	*Not applicable*	*Not applicable*
Employee - unknown group	93 (0.7%)	23	56	14
Not Active - child	1,388 (10.5%)	*Not applicable*	*Not applicable*	*Not applicable*
Not Active - pensioner	1,029 (7.8%)	*Not applicable*	*Not applicable*	*Not applicable*
Not Active – university student	2 (<0.0%)	*Not applicable*	*Not applicable*	*Not applicable*
Not Active - school student	2,345 (17.7%)	*Not applicable*	*Not applicable*	*Not applicable*
Missing Data	*8,347 (62.9%)*			
Grand total	*13,272 (100%)*			

We give ISCO-08 one-digit occupation codes in squared brackets and report how many people are in each occupational category (absolute and relative frequencies). The last three columns show how many referrals, from each occupational category, are employed in the public or private sector (row-wise absolute frequencies).

Table S2A The absolute frequency of referees' jobs by ISCO-08 occupational categories.

ISCO-08 one digit	Cases	ISCO-08 two digits	Cases	ISCO-08 three digits	Cases
Armed forces occupations (0)	19	Armed forces occupations, other ranks (03)	19	Armed forces occupations, other ranks (031)	19
Managers (1)	25	Chief executives, senior officials and legislators (11)	10	Managing directors and chief executives (112)	10
		Administrative and commercial managers (12)	14	Business services and administration managers (121)	11
				Sales, marketing and development managers (122)	3
		Production and specialized services managers (13)	1	Professional services managers (134)	1
Professionals (2)	211	Science and engineering professionals (21)	13	Mathematicians, actuaries and statisticians (212)	1
				Engineering professionals (214)	5
				Architects, planners, surveyors and designers (216)	1
		Health professionals (22)	62	Medical doctors (221)	19
				Nursing and midwifery professionals (222)	23
				Other health professionals (226)	16
		Teaching professionals (23)	57	University and higher education teachers (231)	6
				Secondary education teachers (233)	32
				Primary school and early childhood teachers (234)	17
				Other teaching professionals (235)	2
		Business and administration professionals (24)	16	Finance professionals (241)	6
				Administration professionals (242)	10
		Information and communications technology professionals (25)	9	Software and applications developers and analysts (251)	9
		Legal, social and cultural professionals (26)	53	Legal professionals (261)	22
				Librarians, archivists and curators (262)	2
				Social and religious professionals (263)	15
				Authors, journalists and linguists (264)	13
				Creative and performing artists (265)	1
Technicians and associate professionals (3)	30	Science and engineering associate professionals (31)	2	Physical and engineering science technicians (311)	2
		Health associate professionals (32)	4	Medical and pharmaceutical technicians (321)	1
				Other health associate professionals (325)	3
		Business and administration associate professionals (33)	14	Sales and purchasing agents and brokers (332)	4
				Business services agents (333)	2
				Regulatory government associate professionals (335)	8
		Legal, social, cultural and related associate professionals (34)	5	Sports and fitness workers (342)	5
Clerical support workers (4)	29	General and keyboard clerks (41)	4	General office clerks (411)	1
				Keyboard operators (413)	3
		Customer services clerks (42)	15	Tellers, money collectors and related clerks (421)	6
				Client information workers (422)	9

		Numerical and material recording clerks (43)	8	Material-recording and transport clerks (432)	8
		Other clerical support workers (44)	2	Other clerical support workers (441)	2
Service and sales workers (5)	85	Personal service workers (51)	8	Hairdressers, beauticians and related workers (514)	7
				Other personal services workers (516)	1
		Sales workers (52)	37	Shop salespersons (522)	37
		Personal care workers (53)	5	*Not available*	
		Protective services workers (54)	35	Protective services workers (541)	35
Craft and related trades workers (7)	18	Building and related trades workers, excluding electricians (71)	5	Building frame and related trades workers (711)	5
		Metal, machinery and related trades workers (72)	4	Sheet and structural metal workers, moulders and welders, and related workers (721)	3
				Machinery mechanics and repairers (723)	1
		Handicraft and printing workers (73)	2	Handicraft workers (731)	1
				Printing trades workers (732)	1
		Electrical and electronic trades workers (74)	5	Electrical equipment installers and repairers (741)	5
		Food processing, woodworking, garment and other craft and related trades workers (75)	2	Garment and related trades workers (753)	2
Plant and machine operators, and assemblers (8)	13	Drivers and mobile plant operators (83)	13	Car, van and motorcycle drivers (832)	11
				Heavy truck and bus drivers (833)	1
				Ships' deck crews and related workers (835)	1
Elementary occupations (9)	2	Cleaners and helpers (91)	1	Domestic, hotel and office cleaners and helpers (911)	1
		Labourers in mining, construction, manufacturing and transport (93)	1	Mining and construction labourers (931)	1
Total (valid information)	*432*		*426*		*411*
Active - household (not formally working)	10	Active - household (not formally working)	10	Active - household (not formally working)	10
Active - maternity leave	22	Active - maternity leave	22	Active - maternity leave	22
Active - medical leave	1	Active - medical leave	1	Active - medical leave	1
Active - unemployed	38	Active - unemployed	38	Active - unemployed	38
Employee with no formal job - unknown group	1	Employee with no formal job - unknown group	1	Employee with no formal job - unknown group	1
Employee - unknown group	796	Employee - unknown group	802	Employee - unknown group	817
Not Active - child	107	Not Active - child	107	Not Active - child	107
Not Active - pensioner	537	Not Active - pensioner	537	Not Active - pensioner	537
Not Active - student	22	Not Active - student	22	Not Active - student	22
Not Active - school student	331	Not Active - school student	331	Not Active - school student	331
Missing Data	4,598	Missing Data	4,598	Missing Data	4,598
Grand total	6,895		6,895		6,895

The information is structured following the ISCO-08 structure. We give ISCO-08 occupation codes (one-, two- and three-digits) in brackets and report how many people are in each occupational category.

Table S2B The frequency of referees' jobs by ISCO-08 occupational categories and sector type.

ISCO-08 three digits	Cases	Employed in the public sector	Employed in the private sector	Missing data about type of sector
Armed forces occupations, other ranks (031)	19	19	0	0
Managing directors and chief executives (112)	10	0	9	1
Business services and administration managers (121)	11	1	7	3
Sales, marketing and development managers (122)	3	0	3	0
Professional services managers (134)	1	0	1	0
Mathematicians, actuaries and statisticians (212)	1	1	0	0
Engineering professionals (214)	5	2	3	0
Architects, planners, surveyors and designers (216)	1	0	1	0
Medical doctors (221)	19	14	5	0
Nursing and midwifery professionals (222)	23	20	3	0
Other health professionals (226)	16	1	15	0
University and higher education teachers (231)	6	6	0	0
Secondary education teachers (233)	32	32	0	0
Primary school and early childhood teachers (234)	17	12	5	0
Other teaching professionals (235)	2	2	0	0
Finance professionals (241)	6	1	5	0
Administration professionals (242)	10	9	1	0
Software and applications developers and analysts (251)	9	0	9	0
Legal professionals (261)	22	10	12	0
Librarians, archivists and curators (262)	2	2	0	0
Social and religious professionals (263)	15	2	13	0
Authors, journalists and linguists (264)	13	3	10	0
Creative and performing artists (265)	1	1	0	0
Physical and engineering science technicians (311)	2	1	1	0
Medical and pharmaceutical technicians (321)	1	0	1	0
Other health associate professionals (325)	3	2	1	0
Sales and purchasing agents and brokers (332)	4	1	3	0
Business services agents (333)	2	0	2	0
Regulatory government associate professionals (335)	8	7	1	0
Sports and fitness workers (342)	5	3	2	0
General office clerks (411)	1	1	0	0
Keyboard operators (413)	3	3	0	0
Tellers, money collectors and related clerks (421)	6	0	6	0
Client information workers (422)	9	0	9	0
Material-recording and transport clerks (432)	8	1	7	0
Other clerical support workers (441)	2	2	0	0
Hairdressers, beauticians and related workers (514)	7	0	7	0
Other personal services workers (516)	1	0	1	0
Shop salespersons (522)	37	0	37	0
Protective services workers (541)	35	33	2	0
Building frame and related trades workers (711)	5	0	5	0
Sheet and structural metal workers, moulders and welders, and related workers (721)	3	1	2	0
Machinery mechanics and repairers (723)	1	0	1	0
Handicraft workers (731)	1	0	1	0
Printing trades workers (732)	1	0	1	0

Electrical equipment installers and repairers (741)	5	3	2	0
Garment and related trades workers (753)	2	0	2	0
Car, van and motorcycle drivers (832)	11	0	11	0
Heavy truck and bus drivers (833)	1	1	0	0
Ships' deck crews and related workers (835)	1	0	1	0
Domestic, hotel and office cleaners and helpers (911)	1	0	1	0
Mining and construction labourers (931)	1	0	1	0
Other occupational categories (non-ISCO-08)				
Active - household (not formally working)	10	*Not applicable*	*Not applicable*	*Not applicable*
Active - maternity leave	22	0	4	18
Active - medical leave	1	1	0	0
Active - unemployed	38	*Not applicable*	*Not applicable*	*Not applicable*
Employee with no formal job - unknown group	1	*Not applicable*	*Not applicable*	*Not applicable*
Employee - unknown group	817	224	543	50
Not Active - child	107	*Not applicable*	*Not applicable*	*Not applicable*
Not Active - pensioner	537	*Not applicable*	*Not applicable*	*Not applicable*
Not Active - student	22	*Not applicable*	*Not applicable*	*Not applicable*
Not Active - school student	331	*Not applicable*	*Not applicable*	*Not applicable*
Total unemployed (including unemployable)	1,046	-	-	-
Total employed	1,251	-	-	-
Total employed by type of sector	1,179	*422*	*757*	-
Missing data by type of sector	72	-	-	*72*
Total	2,297	-	-	-

We give ISCO-08 occupation codes (three-digits) in brackets and report how many people are in each occupational category. For each occupational category, we report how many referees work in the public or private sector (row-wise absolute frequencies).

Table S3A The absolute frequency of referrals' jobs by ISCO-08 occupational categories.

ISCO-08 one digit	Cases	ISCO-08 two digits	Cases	ISCO-08 three digits	Cases
Armed forces occupations (0)	2	Armed forces occupations, other ranks (03)	2	Armed forces occupations, other ranks (031)	2
Professionals (2)	31	Science and engineering professionals (21)	1	Electrotechnology engineers (215)	1
		Health professionals (22)	10	Medical doctors (221)	2
				Nursing and midwifery professionals (222)	6
				Veterinarians (225)	1
		Teaching professionals (23)	8	University and higher education teachers (231)	2
				Secondary education teachers (233)	4
				Primary school and early childhood teachers (234)	2
		Information and communications technology professionals (25)	1	Software and applications developers and analysts (251)	1
		Legal, social and cultural professionals (26)	11	Legal professionals (261)	4
				Social and religious professionals (263)	3
				Authors, journalists and linguists (264)	4
Technicians and associate professionals (3)	2	Health associate professionals (32)	1	Other health associate professionals (325)	1
		Business and administration associate professionals (33)	1	Regulatory government associate professionals (335)	1
Clerical support workers (4)	4	General and keyboard clerks (41)	1	General office clerks (411)	1
		Customer services clerks (42)	2	Client information workers (422)	2
		Numerical and material recording clerks (43)	1	Material-recording and transport clerks (432)	1
Service and sales workers (5)	13	Personal service workers (51)	1	Hairdressers, beauticians and related workers (514)	1
		Sales workers (52)	8	Shop salespersons (522)	8
		Personal care workers (53)	1		
		Protective services workers (54)	3	Protective services workers (541)	3
Craft and related trades workers (7)	3	Building and related trades workers, excluding electricians (71)	2	Building frame and related trades workers (711)	1
				Building finishers and related trades workers (712)	1
		Electrical and electronic trades workers (74)	1	Electrical equipment installers and repairers (741)	1
Plant and machine operators, and assemblers (8)	2	Drivers and mobile plant operators (83)	2	Car, van and motorcycle drivers (832)	2
Elementary occupations (9)	2	Cleaners and helpers (91)	1	Domestic, hotel and office cleaners and helpers (911)	1
		Labourers in mining, construction, manufacturing and transport (93)	1	Mining and construction labourers (931)	1
Total (valid information)	59		59		57
Active - household (not formally working)	1	Active - household (not formally working)	1	Active - household (not formally working)	1
Active - maternity leave	4	Active - maternity leave	4	Active - maternity leave	4
Active - unemployed	3	Active - unemployed	3	Active - unemployed	3

Employee with no formal job - unknown group	1	Employee with no formal job - unknown group	1	Employee with no formal job - unknown group	1
Employee - unknown group	93	Employee - unknown group	93	Employee - unknown group	95
Not Active - child	1,388	Not Active - child	1,388	Not Active - child	1,388
Not Active - pensioner	1,029	Not Active - pensioner	1,029	Not Active - pensioner	1,029
Not Active - student	2	Not Active - student	2	Not Active - student	2
Not Active - school student	2,345	Not Active - school student	2,345	Not Active - school student	2,345
Missing Data	8,347	Missing Data	8,347	Missing Data	8,347
Grand total	13,272		13,272		13,272

The information is structured following the ISCO-08 structure. We give ISCO-08 occupation codes in brackets and report how many people are in each occupational category.

Table S3B The frequency of referrals' jobs by ISCO-08 occupational categories and sector type.

ISCO-08 three digits	Cases	Employed in the public sector	Employed in the private sector	Missing data about type of sector
Armed forces occupations, other ranks (031)	2	2	0	0
Electrotechnology engineers (215)	1	0	1	0
Medical doctors (221)	2	1	1	0
Nursing and midwifery professionals (222)	6	5	1	0
Veterinarians (225)	1	0	1	0
University and higher education teachers (231)	2	1	1	0
Secondary education teachers (233)	4	3	1	0
Primary school and early childhood teachers (234)	2	1	1	0
Software and applications developers and analysts (251)	1	0	1	0
Legal professionals (261)	4	1	3	0
Social and religious professionals (263)	3	0	3	0
Authors, journalists and linguists (264)	4	0	4	0
Other health associate professionals (325)	1	0	1	0
Regulatory government associate professionals (335)	1	1	0	0
General office clerks (411)	1	0	1	0
Client information workers (422)	2	0	2	0
Material-recording and transport clerks (432)	1	0	1	0
Hairdressers, beauticians and related workers (514)	1	0	1	0
Shop salespersons (522)	8	0	8	0
Protective services workers (541)	3	3	0	0
Building frame and related trades workers (711)	1	0	1	0
Building finishers and related trades workers (712)	1	0	1	0
Electrical equipment installers and repairers (741)	1	0	1	0
Car, van and motorcycle drivers (832)	2	0	2	0
Domestic, hotel and office cleaners and helpers (911)	1	0	1	0
Mining and construction labourers (931)	1	0	1	0
Other occupational categories (non-ISCO-08)				
Active - household (not formally working)	1	*Not applicable*	*Not applicable*	*Not applicable*
Active - maternity leave	4	0	0	4
Active - unemployed	3	*Not applicable*	*Not applicable*	*Not applicable*
Employee with no formal job - unknown group	1	*Not applicable*	*Not applicable*	*Not applicable*
Employee - unknown group	95	24	57	14
Not Active - child	1,388	*Not applicable*	*Not applicable*	*Not applicable*
Not Active - pensioner	1,029	*Not applicable*	*Not applicable*	*Not applicable*
Not Active - student	2	*Not applicable*	*Not applicable*	*Not applicable*
Not Active - school student	2,345	*Not applicable*	*Not applicable*	*Not applicable*
Total unemployed (including unemployable)	*4,769*	-	-	-
Total employed	*156*	-	-	-
Total employed by type of sector	*138*	*42*	*96*	-
Missing data by type of sector	*18*	-	-	*18*
Total	4,925	-	-	-

We give ISCO-08 occupation codes (three-digits) in brackets and report how many people are in each occupational category. For each occupational category, we report how many referrals work in the public or private sector (row-wise absolute frequencies).

Table S4 The covariate model.

Effects	Covariate model					
	coef	exp(coef)	se(coef)	z	Pr(>|z|)	
Age difference	-0.697	0.498	0.041	-17.081	0.000	***
Avg. age of the referrals	0.004	1.004	0.029	0.156	0.876	
Age of the referee	0.557	1.746	0.034	16.204	0.000	***
Sex difference (males = 1, females = 2)	0.705	2.023	0.034	20.584	0.000	***
Sex of the referrals	0.087	1.091	0.035	2.513	0.012	*
Sex of the referee	0.046	1.047	0.020	2.260	0.024	*
Referral in public sector	-0.347	0.707	0.083	-4.174	0.000	***
Referee in public sector	-0.035	0.965	0.015	-2.324	0.020	*
Referral in medical sector	-0.181	0.835	0.092	-1.977	0.048	*
Referee in medical sector	0.000	1.000	0.014	0.096	0.995	
Active workforce difference (Active = 1, non-active = 0)	0.167	1.182	0.047	3.552	0.000	***
Active referrals	-0.843	0.430	0.050	-16.999	0.000	***
Active referees	0.827	2.286	0.039	21.119	0.000	***
AIC	9,375.042					
Num. events	1,179					
Num. obs.	1,250,975					

Signif. Codes: 0 ‘***’ 0.001 ‘**’ 0.01 ‘*’ 0.05 ‘.’ 0.1 ‘ ’ 1

Table S5 The network model.

	Network model					
Effects	coef	exp(coef)	se(coef)	z	Pr(>|z|)	
Individual contact popularity (the in-degree of the referral)	-5.139	0.006	0.309	-16.654	0.000	***
Joint contact popularity	0.110	1.116	0.016	6.773	0.000	***
Reciprocation	0.123	1.131	0.009	13.439	0.000	***
In-degree of the referee	-1.472	0.229	0.084	-17.470	0.000	***
Out-degree of the referral	-2.880	0.056	0.195	-14.744	0.000	***
Nominations among contacts	0.074	1.077	0.007	10.464	0.000	***
Shared referee	0.104	1.110	0.010	10.709	0.000	***
AIC	8592.55					
Num. events	1179					
Num. obs.	1250975					

Signif. Codes: 0 '***' 0.001 '**' 0.01 '*' 0.05 '.' 0.1 ' ' 1

Table S6 The joint model.

	Joint model					
Effects	Coef	Exp(coef)	SE(Coef)	z	Pr(>|z|)	
Age difference	-0.652	0.521	0.045	-14.635	0.000	***
Avg. age of the referrals	0.019	1.020	0.031	0.638	0.524	
Age of the referee	0.477	1.612	0.038	12.514	0.000	***
Sex difference (males = 1, females = 2)	0.628	1.873	0.038	16.376	0.000	***
Sex of the referrals	0.107	1.113	0.040	2.639	0.008	**
Sex of the referee	0.039	1.040	0.021	1.833	0.067	.
Referral in public sector	-0.284	0.753	0.085	-3.327	0.001	***
Referee in public sector	-0.044	0.957	0.016	-2.766	0.006	**
Referral in medical sector	-0.130	0.878	0.089	-1.464	0.143	
Referee in medical sector	-0.003	0.997	0.014	-0.211	0.833	
Active workforce difference (Active = 1, non-active = 0)	0.226	1.253	0.054	4.204	0.000	***
Active referrals	-0.707	0.493	0.058	-12.107	0.000	***
Active referees	0.822	2.276	0.043	19.245	0.000	***
Individual contact popularity (the in-degree of the referral)	-4.878	0.008	0.306	-15.937	0.000	***
Joint contact popularity	0.104	1.110	0.022	4.745	0.000	***
Reciprocation	0.105	1.111	0.010	10.078	0.000	***
In-degree of the referee	-1.182	0.307	0.088	-13.395	0.000	***
Out-degree of the referral	-2.655	0.070	0.194	-13.713	0.000	***
Nominations among contacts	0.078	1.081	0.007	10.530	0.000	***
Shared referee	0.099	1.104	0.009	10.513	0.000	***
AIC	6293.281					
Num. events	1179					
Num. obs.	1250975					

Signif. Codes: 0 ‘***’ 0.001 ‘**’ 0.01 ‘*’ 0.05 ‘.’ 0.1 ‘ ’ 1

Table S7 Relational hyperevent model assessment: comparing the covariate, the network, and the joint models, with random imputation of sex missing data.

	Covariate model			Network model			Joint model		
	exp(Coef)	Coef (SE)		exp(Coef)	Coef (SE)		Exp(Coef)	Coef (SE)	
Age difference	0.499	-0.695 (0.041)	***				0.514	-0.665 (0.045)	***
Avg. age of the referrals	1.069	0.067 (0.028)	*				1.057	0.055 (0.030)	.
Age of the referee	1.870	0.626 (0.034)	***				1.788	0.581 (0.038)	***
Sex difference (males = 1, females = 2)	1.069	0.067 (0.033)	*				1.034	0.033 (0.036)	
Sex of the referrals	1.023	0.023 (0.033)					1.013	0.013 (0.036)	
Sex of the referee	0.984	-0.016 (0.030)					0.990	-0.010 (0.033)	
Referral in public sector	0.731	-0.314 (0.082)	***				0.802	-0.221 (0.083)	**
Referee in public sector	0.959	-0.042 (0.015)	**				0.949	-0.052 (0.016)	***
Referral in medical sector	0.846	-0.167 (0.089)	.				0.879	-0.129 (0.092)	
Referee in medical sector	1.004	0.004 (0.013)					1.009	0.009 (0.014)	
Active workforce difference (Active = 1, non-active = 0)	1.224	0.202 (0.047)	***				1.290	0.255 (0.054)	***
Active referrals	0.480	-0.734 (0.051)	***				0.528	-0.638 (0.060)	***
Active referees	3.004	1.100 (0.037)	***				2.790	1.026 (0.041)	***
Individual contact popularity (the in-degree of the referral)				0.006	-5.036 (0.300)	***	0.008	-4.813 (0.293)	***
Joint contact popularity				1.052	0.051 (0.007)	***	1.051	0.050 (0.007)	***
Reciprocation				1.132	0.124 (0.010)	***	1.119	0.112 (0.010)	***
In-degree of the referee				0.229	-1.473 (0.084)	***	0.282	-1.267 (0.089)	***
Out-degree of the referral				0.062	-2.785 (0.193)	***	0.079	-2.540 (0.194)	***
Nominations among contacts				1.064	0.062 (0.005)	***	1.064	0.062 (0.006)	***
Shared referee				1.112	0.106 (0.009)	***	1.107	0.102 (0.009)	***
AIC	9806.05			8605.85			6596.82		
Num. events	1179			1179			1179		
Num. obs.	1248665			1248665			1248665		

Signif. codes: 0 '***' 0.001 '**' 0.01 '*' 0.05 '.' 0.1 ' ' 1.
